# Supplementary material for: Chromothripsis during telomere crisis is independent of NHEJ, and consistent with a replicative origin
Source: Genome Res. 2019 May;29(5):737–49. doi: 10.1101/gr.240705.118 (PMC6499312; doi:10.1101/gr.240705.118)
Supplement: Supplemental Material [file supp_gr.240705.118_Supplemental_file_1.zip › contigs/annotated_contigs/DB53/contig.3.DB53_length_863_mean_cov_10.0787949015.docx]

**DB53_length_863_mean_cov_10.0787949015**

```
GGAAAAAACCAGAAGTGCTTTCTCCTCTCACATTCCACTCAACACTTCTCTCACCAAATGTGTGTGGGGATTTCTCTCCACCAACAACC
 >chr8:135808539-135808766 - E=1e-124
AGCAGTTCTTTACTGCAGATTCTCCAGTGGATACCAGCCAGGTGTCCTCTAATTCAGTTCAATTCTGACATTATCTACTGGGAGATAGT

GTCAGATCCCGCAAGTGGAGGGCTCAGTCCTACAAGACTACCCCTTA|CT|CACATGAGCTTGGAAGCAAAATAAATTGAAATAGCACT
                                                >chr8:135807092-135807412 + E=1e-180
TACTAAACAACTTTGTTGCTAGCACTGTGCAGGGTCCTTCACATAGTTCTCTTATCTAGTCCTCCTAACAACCTGATAGCCACCATTTT

TTTATTTTAATTTTTTTAACAGAGAACTATCCAGAAAAGATGAACAACTTGTTCAAGGTCACAAAAATGGCAAGTGAGGGAGCCTAGAT

TAGATCAGAGGTCTAATTCCCAAGGGTATGTTTGTGATATTGTGAAATATATATTTGCTCTTCATCTCCATTTCCTAAAGCCCTTGGAA

TCTCTGAAATTGT|CCT|TCTTTAAAAAAATTAAACTGAGGACAGGAAGGTAAATTAAAGGGGAGATTTGGAGACTTGTGGTAACTGGT
                 >chr8:135818570-135818885 - E=1e-177
TTATTGCAACTTAATGTGCAGCATCCTCATTAGTCAAGTATCATGTGCCTACCTAGGCATGAAATAGCTATTTTTTTCCTGATTTAAAT

CTGGAAAGTTTCAGTTGTAGTACAGTTTGAATAGTTGTTGTTGTAAATAGCCACTGGACTTTTTAAAAAAGTATTTTTCTTCAAAAACA

GTGAAGGTCATCTTGTATCCATAGAGACCACATGTACTTTCCTCATTATTTCCAGCATTATTTATA
```
